# Advances in the Early Warning of Shellfish Toxification by *Dinophysis acuminata*

**DOI:** 10.3390/toxins16050204

**Published:** 2024-04-24

**Authors:** Alexandra Duarte Silva, Susana Margarida Rodrigues, Lia Godinho

**Affiliations:** 1 IPMA—Instituto Português do Mar e da Atmosfera, Rua Alfredo Magalhães Ramalho, 6, 1495-165 Lisboa, Portugal; srodrigues@ipma.pt; 2 ICNF—Instituto de Conservação da Natureza e Florestas, Av. da República, 16, 1050-191 Lisboa, Portugal; lia.godinho@icnf.pt

**Keywords:** *Dinophysis*, diarrhetic shellfish toxins, okadaic acid, time series, generalized additive models, Tweedie distribution, early warning, monitoring, food safety, HABs

## Abstract

In Western Europe, the incidence of DST is likely the highest globally, posing a significant threat with prolonged bans on shellfish harvesting, mainly caused by species of the dinoflagellate genus *Dinophysis*. Using a time series from 2014 to 2020, our study aimed (i) to determine the concentration of *D. acuminata* in water at which shellfish toxin levels could surpass the regulatory limit (160 µg OA equiv kg^−1^) and (ii) to assess the predictability of toxic events for timely mitigation actions, especially concerning potential harvesting bans. The analysis considered factors such as (i) overdispersion in the data, (ii) distinct periods of presence and absence, (iii) the persistence of cells, and (iv) the temporal lag between cells in the water and toxins in shellfish. Four generalized additive models were tested, with the Tweedie (TW-GAM) model showing superior performance (>85%) and lower complexity. The results suggest existing thresholds currently employed (200 and 500 cells L^−1^) are well-suited for the Portuguese coast, supported by empirical evidence (54–79% accuracy). The developed algorithm allows for thresholds to be tailored on a case-by-case basis, offering flexibility for regional variations.

## 1. Introduction

The term harmful algal blooms (HABs) refers to any proliferation of microalgae perceived as harmful owing to its negative impact on public health, the economy (e.g., aquaculture activities, tourism) and/or on the environment, due to the production of toxins (regardless of the concentration) or by attaining high biomass [1]. HABs cannot easily be eliminated or prevented, but the potentially negative consequences can be managed and mitigated, which is particularly valuable in aquaculture production systems. According to the last Global Harmful Algal Bloom Status Report (GHSR), across the globe, events associated with seafood biotoxins (1985–2018) accounted for 48% of the total number of events (*n* ~ 9500), followed by high biomass proliferations and/or water discoloration causing a socioeconomic impact (43%), mass animal or plant mortalities (7%), and 2% of other events (including foam and mucilage production) [2]. Among all of the events linked with seafood toxin syndromes, diarrhetic shellfish toxins (DSTs) were ranked second, accounting for 30% of events, immediately below paralytic shellfish toxins (35%) [2]. Western Europe likely has the highest incidence of DSTs in the world [3,4,5,6] and this syndrome is the most harmful in terms of the duration of shellfish harvesting bans [3]. Between 1985 and 2018, a 4-fold increase was reported globally for the main causative species of DSP (belonging to the dinoflagellate genus *Dinophysis*) with impacts consisting mostly of shellfish harvesting closures [7]. Two monitoring strategies can be applied: (1) monitoring the presence of toxins in shellfish, which is mandatory for shellfish consumption and (2) monitoring the presence of toxic phytoplankton species. The first strategy operates according to European Union directives that specify the regulatory level (RL) of 160 µg OA equiv kg^−1^ of shellfish meat of the edible product [8]; mussels (*Mytilus* spp.) are extensively employed as sentinel species in marine biotoxin monitoring programs, primarily owing to their higher toxin accumulation and depuration rates compared to other filter bivalve species [9,10,11,12,13]. The second strategy is based on the definition of thresholds for bloom concentration that may cause shellfish toxicity (warning levels). As the occurrence of toxic cells in water typically precedes the detection of biotoxins, collecting data on the abundance of toxin-producing phytoplankton can offer insights into the likelihood of biotoxin accumulation in bivalves. These numerical limits for toxigenic cell levels are set according to geographic region and are non-regulatory values. They serve as informal technical guidance to assist federal, state, and local authorities, and managers of public or community water systems, to protect public health and harvesting practices. Both strategies are crucial to ensure a thorough assessment of the risk associated with elevated toxin levels in each harvesting area. 

Along the Portuguese coast (NW Atlantic), the main cause of shellfish harvesting bans are those associated with proliferations of *Dinophysis* species, mainly *D. acuminata* and *D. acuta*. These dinoflagellates are producers of the okadaic acid (OA) group of toxins. *Dinophysis* species are usually observed in the water during periods of thermohaline stratification between moderate pulses of upwelling, or during downwelling events [14] and contamination with OA and its analogues ranks amongst the top ten highest levels reported worldwide for bivalves [15]. In Portuguese shellfish harvesting areas, closures are prevalent for almost the entire year, extending from February to November [16,17,18]. These closures are primarily linked to the presence of the parent toxin OA and the occurrence of *D. acuminata* in the water. Additionally, in late summer to autumn, closures can also be attributed to the presence of its analogue dinophysistoxin-2 (DTX2) due to the presence of *D. acuta* [11,19,20]. In Portugal, the current reference levels for *Dinophysis* cells in the water were established through consensus among European monitoring laboratories [21]. These levels are defined at 200 cells L^−1^, a warning threshold indicating potential bloom development, and 500 cells L^−1^, a warning threshold for a potential shellfish harvesting ban due to concentrations that can lead to diarrhetic shellfish poisoning (DSP) events.

Commercially available bivalves for human consumption in Portugal are predominantly sourced from natural banks, particularly in locations such as Ria Formosa on the south coast of the Algarve region. The annual bivalve catch in 2019 reached approximately 6700 tons, with clams accounting for 26.9%, oysters for 13.2%, and *Mytilus* species for 5.5% [22,23]. Wild blue mussels, including *Mytilus galloprovincialis* and/or *M. edulis*, are traditionally handpicked from rocky surfaces and manmade structures. In recent years, the growth of aquaculture production areas along the Portuguese coast, primarily limited to the Algarve coast (south), has facilitated the establishment of offshore companies for mussel production, taking advantage of the region’s favorable oceanographic conditions [24]. On the other hand, for *Donax* clams, a portion of the commercially harvested specimens in the southwest and south coasts of Portugal is obtained through hand dredging. Blue mussels and donax clams are the bivalves more easily accessible for recreational harvest. From 1998 to 2012, around two hundred cases of human intoxication were reported in Portugal due to the consumption of mussels and clams contaminated with DST [11,25,26,27]. When several shellfish species are growing in the same production area and toxin accumulation rates are available, the species with the highest rate may be used as an indicator species. Since 2002, the Marine Biotoxins Laboratory at IPMA has adopted *Mytilus galloprovincialis* and *Donax trunculus* as indicator species for DST [11,20,28].

The aims of this study, performed by analyzing time series data from *D. acuminata* cells in water and OA levels in shellfish from 2014 to 2020, were to determine: (i) the concentration of *D. acuminata* in water at which toxin levels in shellfish could meet or exceed the regulatory limit (160 µg OA equiv kg^−1^) and (ii) the extent to which toxic events can be predicted in time, to inform mitigating actions, most often compromising potential harvesting bans. Furthermore, the model outputs are expected to provide insights that can guide the development of regional early warning thresholds according to shellfish species/habitat.

## 2. Results

### 2.1. Cells in the Water vs. Toxins in Shellfish

**Figure 1:**
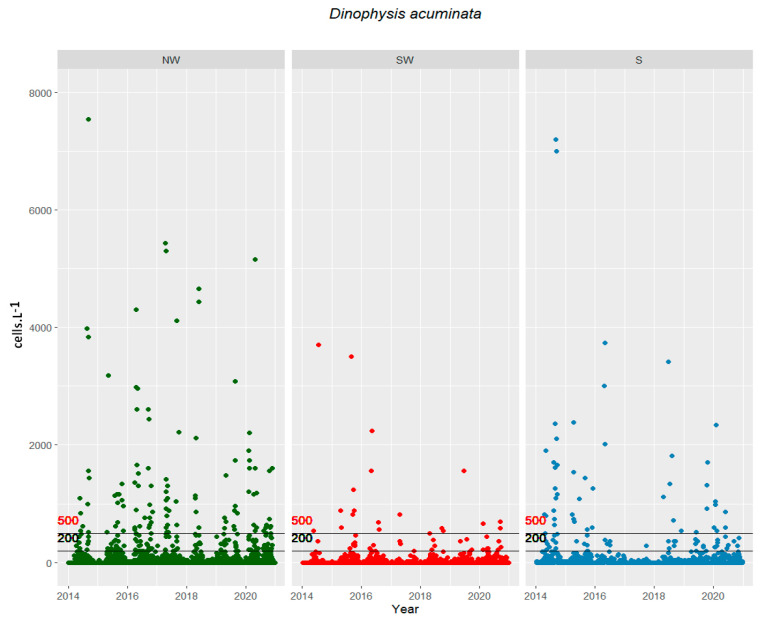
*Dinophysis acuminata* (cells L^−1^) along the NW, SW, and S coasts from 2014 to 2020. The lines on the graph represent the warning (200 cells L^−1^) and closure (500 cells L^−1^) thresholds for phytoplankton for management measures in shellfish production areas.

*D. acuminata* was consistently detected in monitoring samples, primarily from March to October (Figure 1). Concentrations in the water varied significantly by month and between years, with the highest blooms recorded on the northwestern (NW), southwestern (SW), and southern (S) coasts, at levels of about 13 × 10^3^ cells L^−1^ (April 2020), 4 × 10^3^ cells L^−1^ (July 2014), and of 12 × 10^3^ cells L^−1^ (February 2020), respectively. Bloom densities surpassing 200 cells L^−1^ were predominantly observed in the spring and summer (Table 1 and Figure 1). The occurrence of cells in the water exceeding warning levels (200 and 500 cells L^−1^) was more frequent on the northwest coast. These events persisted for a minimum of one month, defined as a period when cell proliferation leads to a harvesting ban due to shellfish toxification. There were consistently more than 20 events annually, each usually lasting several weeks, as illustrated in Figure 1.

**Figure 2:**
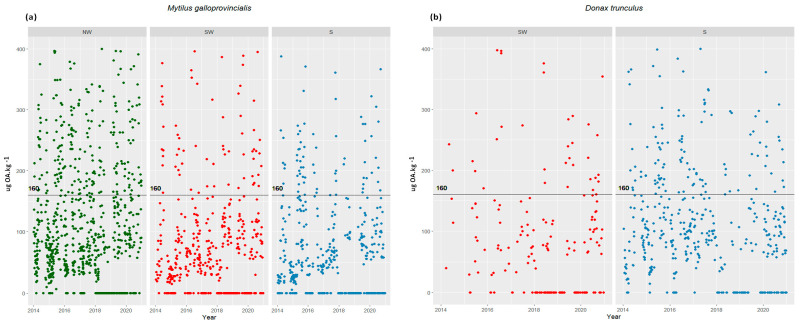
DST concentrations in (**a**) *Mytilus* samples from the NW, SW, and S coast and (**b**) *Donax* from the SW and S coast, between 2014 and 2020. The line on the graph represents the EU RL for DST. (Concentrations above 400 µg OA equiv kg^−1^ are not presented in the graphic because they were discarded from the analysis).

From 2014 to 2020, DSTs were monitored on a weekly basis and consistently detected in shellfish samples from all three coasts (NW, SW, S) throughout the year (Figure 2). The more intense DST episodes typically occurred between March and December. Concentrations exceeding the legal threshold for closure (160 µg OA equiv kg^−1^ of shellfish-derived edible product), leading to harvesting bans, were most prevalent towards the end of spring and during the summer months. Some of these events exhibited exceptionally high concentrations in shellfish, resulting in prolonged periods of shellfish bans (Figure 2 and Table 1). Compared to the SW and S coasts, the NW coast experienced the most significant impact from DST events, especially in 2017 and 2018, when mussels reached particularly high concentrations (Table 1).

### 2.2. Model Fitting

Four generalized additive models, Poisson (POIS-GAM), Tweedie (TW-GAM), zero- inflated Poisson (Z-GAM), and negative binomial (NB-GAM), were tested to explain the relationship between *D. acuminata* cells in the water and the concentration of okadaic acid in *M. galloprovincialis* and *D. trunculus*.

Initially, the data set (*n* = 6463) was examined through a Spearman’s correlation (a non-parametric measure that explores the correlation between variables without assuming a linear relationship and using the ranks of the data for calculation [29]) without lags between cells and toxins and with lags of several weeks (Table 2). The results showed clearly that the monotonic relationship between the variables is weak. Lags greater than 2 weeks did not retrieve any meaningful information (not shown). These results led to further adjustments of the model, such as considering the persistence of cells in the water (>100 cells L^−1^) for at least 2 weeks and a sub-dataset of values below 400 µg OA kg^−1^ (*n* = 4847).

**Figure 3:**
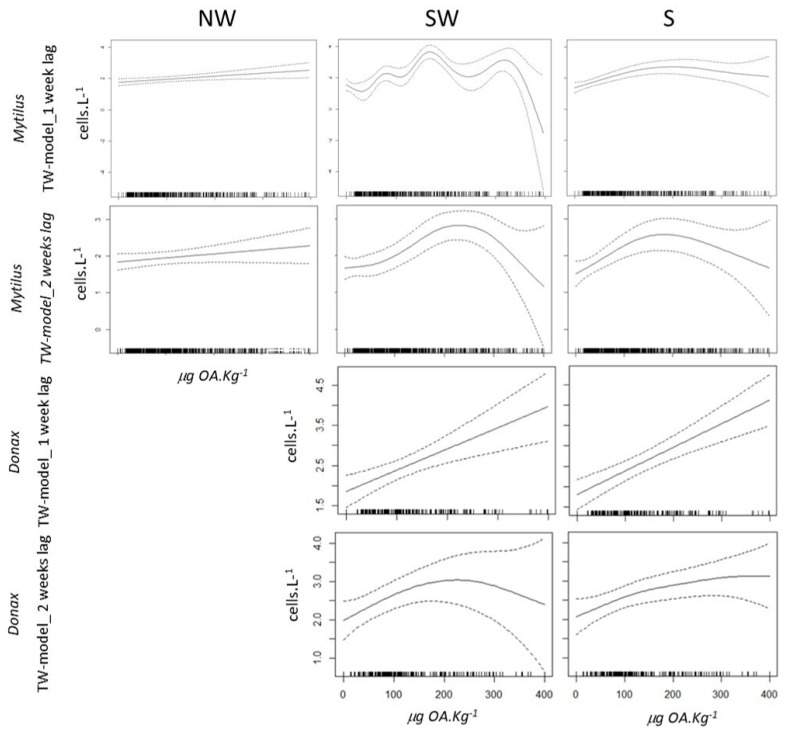
Forms of smoothing functions for the selected covariates for each TW-GAM model. The *y*-axis reflects the logarithm of cell concentration in cells L^−1^, while the *x*-axis represents OA concentration in µg OA kg^−1^. In each plot, the solid line represents the smooth function estimate, and the dashed lines along it denote approximate 95% confidence intervals.

Generally, for both species and both 1- and 2-week lags, the residual plots and the smoothing functions of each fitted model showed an accumulation of residuals at zero (which indicates that, on average, the models tend to predict values close to the observed ones) but with a high degree of variability (overdispersion of positive values) and less significance for the TW-GAM model (Figure 3—forms of smoothing functions).

The selected algorithm represents a generalized additive model with a Tweedie family distribution, using a logarithmic link function, and fitted using the REML estimation method, to analyze the relationship between *Dinophysis acuminata* cells concentration and other predictor variables. It unfolds as follows:

(a) *M. galloprovincialis*_1-week lag
  $$Gam(Dinophysis\;acuminata\;cells\;concentration\sim s(OA\;toxin\;concentration\;in\;M.\;galloprovincialis\;1-\;week\;lag,\;by=Coastal\;area)+Cells\;persistence\;in\;the\;water,\;family=tw\left(link=\log\right),\;method=REML)$$
(b) *M. galloprovincialis*_2-week lag
  $$Gam(Dinophysis\;acuminata\;cells\;concentration\sim s(OA\;toxin\;concentration\;in\;M.\;galloprovincialis\;2-\;week\;lag,\;by=Coastal\;area)+Cells\;persistence\;in\;the\;water,\;family=tw\left(link=\log\right),\;method=REML)$$
(c) *D. trucullus*_1-week lag
  $$Gam(Dinophysis\;acuminata\;cells\;concentration\sim s(OA\;toxin\;concentration\;in\;D.\;trunculus\;1-\;week\;lag,\;by=Coastal\;area)+Cells\;persistence\;in\;the\;water,\;family=tw\left(link=\log\right),\;method=REML)$$
(d) *D. trucullus*_2-week lag
  $$Gam(Dinophysis\;acuminata\;cells\;concentration\sim s(OA\;toxin\;concentration\;in\;D.\;trunculus\;2-\;week\;lag,\;by=Coastal\;area)+Cells\;persistence\;in\;the\;water,\;family=tw\left(link=\log\right),\;method=REML)$$

The quantile–quantile (QQ) plots of the models (to check the fit of a dataset to a specific distribution, assess model assumptions, and identify potential issues such as outliers) showed that only the residuals of the TW model appear to be close to a straight line, suggesting a reasonable normal distributional assumption. The residuals vs. linear predictor plots suggests that the variance is approximately constant as the mean increases for all models with the exception of the NB model, where the constant variance assumption is clearly untenable. The histogram of residuals displayed a relatively symmetric bell-shaped distribution that is evenly centered around zero. This suggests that the normality assumption of variance is likely valid for all the models, particularly for the TW model (with the exception of the NB model). This indicates the model is capturing the underlying patterns in the data. As the predicted values increase, the observed responses tend to follow the same trend, increasing in a linear fashion. This positive linear relationship implies that the model is capturing the underlying patterns in the data effectively and is making predictions that align well with the actual outcomes. However, for cell counts above 500, the models tend to underestimate the real values. The NB model in particular tends to underestimate the actual values and, for higher counts, it tends to overestimate them. In general, the majority of the models showed some overdispersion meaning the variability in the data is greater than what the model predicts.

The global evaluation of the models showed that the TW-GAM models performed better (86–88%) with the lower AIC score (Table 3); POIS-GAM had the highest AIC and the worst performance. The Z-GAM model had the highest deviance explained and the NB-Model the lowest. In general, the Z-GAM model presented better scores in the other performance parameters, such as RMSE (average prediction error, how close the model’s predictions are to the actual values). However, TW-GAM presents good scores as well, characterized by lower complexity compared to the Z-GAM model, making it the optimal choice for fitting the data and consequently adopted as the model of choice. All models have lower accuracy for *Donax* (smaller dataset) than for *Mytilus*.

### 2.3. Model Validation

**Figure 4:**
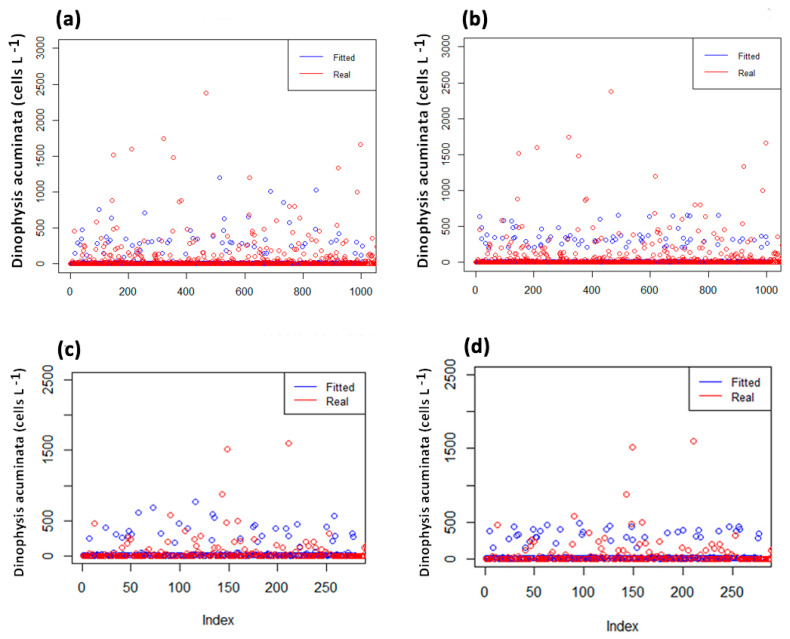
Model validation. GAM–Tweedie. Fitted (blue) vs. real data (red) for (**a**) *Mytilus*_1-week-lag, (**b**) *Mytilus*_2weeks-lag, (**c**) *Donax*_1-week-lag, (**d**) *Donax*_2weeks-lag.

Model validation was performed using a new dataset (*n* = 1616), representing 25% of the dataset of this study, and generated by computer. The model validation indicated higher accuracy of the TW-GAM (54–79%) compared to the other models (Figure 4, Table 3), with the POIS-GAM model exhibiting the lowest scores. Z-GAM had the deviance explained at 100%, which means the model was able to account for and capture all the variability present in the data but with a low performance score (e.g., due to overfitting of the training data). Considering the performance assessment, the TW-GAM model represented the simplest approach and demonstrated consistently high scores, making it the optimal choice for fitting these data and consequently adopted as the primary model in this study. Only the results of this model are presented and discussed, information on the remaining models is available as Supplementary Materials.

Model accuracy is defined as the number of classifications a model correctly predicts divided by the total number of predictions made.

*Mytilus galloprovincialis*—1-week lag model

The TW-GAM employing a one-week lag smooth function exhibited an almost linear rise in cell concentrations with increasing OA levels along the NW coast. Along the SW coast, cell concentrations displayed an initial fluctuating pattern with the rise in OA until 350 µg OA kg^−1^, followed by a sharp decline, accompanied by an increase in standard error (Figure 3). Conversely, the S coast revealed a positive trend in *D. acuminata* cell concentration with increasing OA concentration up to 200 µg OA kg^−1^, followed by a slight decrease and an associated increase in standard error (Figure 3).

The TW-GAM, boasting a model accuracy of 79.4% for a 1-week lag, predicted that concentrations of *D. acuminata* leading to OA accumulation in *M. galloprovincialis* exceeding regulatory limits would be, for the NW coast, 248 ± 25 cells L^−1^; for the SW coast, 447 ± 87 cells L^−1^; and for the S coast, 1128 ± 206 cells L^−1^.

*Mytilus galloprovincialis*—2-week lag model

The TW-GAM, incorporating a two-week lag smooth function, revealed an almost linear escalation in cell concentrations with increasing OA levels along the NW coast (as with a 1-week lag) (Figure 3). Conversely, the SW coast exhibited a nearly linear rise in cell concentrations with increasing OA up to 250 µg OA kg^−1^ (lower than with a 1-week lag), followed by a sharp decline, accompanied by an increase in standard error (as with a 1-week lag) (Figure 3). The S coast displayed a similar trend, albeit with a smoother decrease after 250 µg OA kg^−1^ and an associated increase in the standard error.

With a model accuracy of 54.3% for a 2-week lag, the TW-GAM predicted that concentrations of *D. acuminata* leading to OA accumulation in *M. galloprovincialis* beyond the regulatory limit would be, for the NW coast, 296 ± 26 cells L^−1^; for the SW coast, 505 ± 101 cells L^−1^; and for the S coast, 482 ± 84 cells L^−1^.

*Donax trunculus*—1-week lag model

The TW-GAM, featuring a one-week lag smooth function, demonstrated a strictly linear augmentation in cell concentrations with the increase in OA, observed consistently along both the SW and S coasts. This trend, however, was accompanied by an increase in the standard error (Figure 3).

With a model accuracy of 78.7% for 1-week lag, the TW-GAM forecasted that concentrations of *D. acuminata* leading to OA accumulation in *D. trunculus* surpassing regulatory limits would be, for the SW coast, 334 ± 44 cells L^−1^, and for the S coast, 344 ± 37 cells L^−1^.

*Donax trunculus*—2-weeks lag model

The TW-GAM, incorporating a two-weeks lag smooth function for the SW coast, depicted an almost linear elevation in cell concentrations with the rise of OA until 250 µg OA kg^−1^, followed by a smooth decline thereafter, albeit with an increase in standard error (Figure 3). Conversely, along the S coast, there was an almost linear increase in cell concentrations with the escalation of OA until 300 µg OA kg^−1^, stabilizing thereafter, accompanied by an increase in the standard error (Figure 3).

With a model accuracy of 60% for a 2-week lag, the TW-GAM forecasted that concentrations of *D. acuminata* leading to OA accumulation in *D. trunculus* exceeding regulatory limits would be, for the SW coast, 400 ± 85 cells L^−1^, and for the S coast, 350 ± 48 cells L^−1^.

## 3. Discussion

A major challenge faced by shellfish monitoring programs is to succeed in raising awareness among the health and fishery authorities of their respective countries as regards the nature of the problem and the need to establish early warning systems. Such programs will contribute (i) to improving the management of toxic events, which have serious consequences, including severe illness and the loss of human life and (ii) to designing contingency plans to mitigate the impact of toxic events on shellfish resources, small-scale fishing, and tourism [18,30].

Shellfish toxification typically is a function of the accumulation of toxins in the resource and their presence is determined by a complex balance between food selection, adsorption, species-specific enzymatic transformations, allometric processes [3,9,31], and is influenced by the HAB species, various environmental factors (e.g., temperature, salinity, nutrient levels, sunlight), and geographical location [32]. Variations in the predicted shellfish toxin values for *Mytilus*, *Donax*, and different areas (NW, SW, S) were anticipated and have been previously elucidated here (Section 4.1). Discrepancies between the values for toxicity in bivalves and the abundance of microalgae were also observed and actually anticipated since the toxicity value in bivalves reflects continuous exposure before sample collection, whereas the plankton toxin value represents an instantaneous determination at the time of capture, prior to accumulation in bivalves. Even in cases where bivalve species coexist in the same region and are exposed to the same bloom, significant differences in toxin accumulation can occur [3,31]. However, in relation to mussel and donax species, the DST results over the seven-year study period indicated that in coastal production areas where both species coexisted (L5, L6—SW, and L8—S), both exhibited similar toxin accumulation dynamics, leading to notably high DST concentrations.

GAM models are particularly useful for identifying threshold response levels common in ecological systems and were chosen instead of GLM because they do not assume a priori any specific form of the dependent and predictive variable relationships. They are for uncovering and estimating nonlinear effects, especially in zero-abundant datasets, being based on the assumption that the data structure is “additive” and that the relationship between covariates is likely to be smooth [33,34]. This additive structure of TW-GAM models facilitated our interpretation of the contributions of individual predictors (e.g., OA concentration, persistence) to the dependent variable (*D. acuminata*), and our understanding of the relationships between variables. The algorithm was refined to take into account the persistence of cells in the water, along with seasonality, as a predictive variable. The persistence of cells served as an indicator of sustained favorable conditions leading to significant changes in cell numbers until warning levels were attained (200 cells L^−1^). Considering seasonality involves recognizing the occurrence of zeros is not random but follows a regular, recurring pattern associated with seasons. The model was adjusted to account for these seasonal patterns, ensuring a more accurate representation of key processes as they occurred during particular months or under particular conditions. Seasonal adjustments improved the overall understanding of the data. The TW-GAM model demonstrated effectiveness and flexibility in dealing with the seasonality and discrepancies between cells in the water and the subsequent toxification of shellfish (performance scores ranging between 85.98 and 87.86%) [35]. TW-GAM performed well in accurately predicting relatively low cell concentrations of *D. acuminata* (<500 cells L^−1^), leading to a high incidence of shellfish contaminated with DST toxins, mainly by high concentrations of OA [36]. Correlations with one- and two-week lag maximums reveled the consistency of a week of bivalve exposure to cells in the water before toxicity reaches the closure level. The model with a one-week lag consistently yielded an accuracy of 79%, performing better than the two-week lag model. Thresholds for *Donax* were always lower than for *Mytilus*, which infers that clams toxify faster than mussels under the conditions described herein for the SW and S coasts. The smaller dataset available for *Donax* may also influence these scores despite the fitting and validation performed well for lower concentrations. On the other hand, *Mytilus* thresholds increased towards the south coast, which may reflect the lower phytoplankton abundances and a shorter productive period known in these areas [37,38].

This high explanatory power may be attributed, in our opinion, to the incorporation of cell persistence, the inclusion of lags in correlations, the focusing of the model on a concise dataset within the time series, and the accounting for various shellfish species. Other authors using GAM models to understand *Dinophysis* dynamics by correlating environmental parameters obtain scores ≤ 60% (32 and references therein). This study showed for the first time that the currently employed alert thresholds are well-suited for the Portuguese coast and are substantiated by empirical evidence rather than consensus. Predicted values for cells in the water were generally between the range of the current guidance thresholds (200 and 500 cells L^−1^), irrespective of the coast orientation and shellfish species. With the implementation of this algorithm, there is now an opportunity to explore tailored thresholds on a case-by-case basis for specific areas or regions, potentially allowing for the strategic/targeted use of safer higher levels if required. Hindcast simulations demonstrated the efficacy of the new thresholds, showcasing a notably acceptable performance. It is still necessary to evaluate if these changes need to be translated into modifications to the current risk assessment and management actions adopted by the bivalve sector and regulatory authorities. The definition of thresholds, in particular those that anticipate the potential toxicity of seafood products, is widely recognized a harvesting management tool.

## 4. Materials and Methods

### 4.1. Study Area

**Figure 5:**
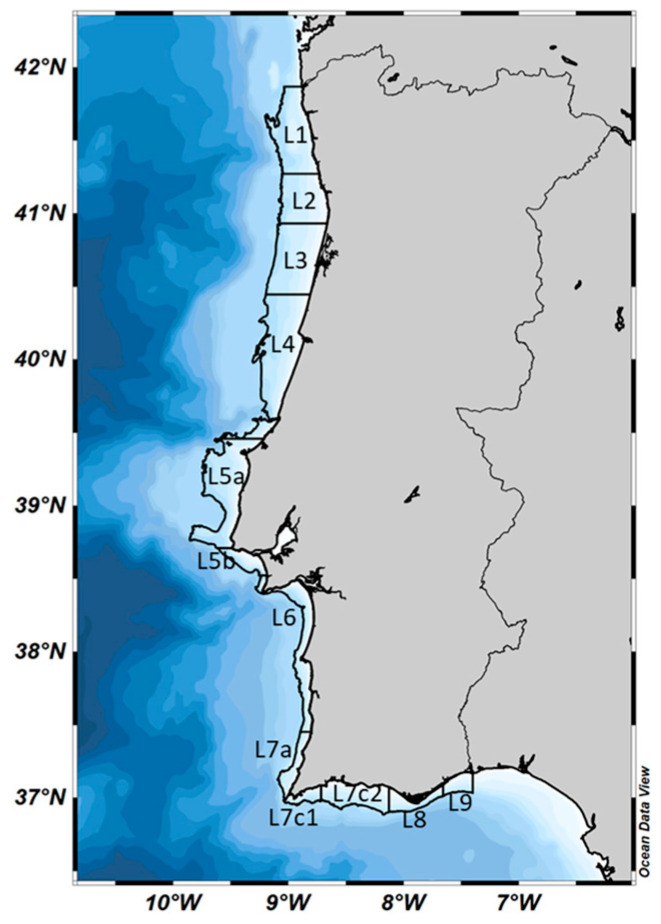
Portuguese shellfish production areas. Areas delimited with “L” represent the thirteen coastal production areas and are numbered (L1, L2, L3, L4, L5a, L5b, L6, L7a, L7c1, L7c2, L7c2, L8 and L9) Source: National Monitoring Program of Shellfish Molluscs (SNMB) held by IPMA [39].

In this analysis, thirteen classified coastal harvesting areas (L1 to L9) [39], distributed along the Portuguese coast, were grouped into three primary regions, northwestern (NW, L1 to L4), southwestern (SW, L5 to L7a), and southern (S, L7c1 to L9) (Figure 5). This aggregation reflected the geographical and oceanographic diversity inherent in this extensive region. Its geography can be briefly summarized as a continental margin divided into sub-regions (NW, SW, S) by the occurrence of seamounts, submarine canyons (especially along the NW coast), and abyssal plains.

The NW sector is characterized by a wider shelf (35–60 km) and a gentler gradient than the SW (10–20 km) and southern (8–28 km) areas [37]. From an oceanographic point of view, the Portuguese coast is located in a biogeographic transition zone, between temperate and subtropical waters under the influence of the Iberian upwelling system [40]. A large fraction of its variability is forced by winds and freshwater input from river runoff. Seasonal and interannual changes are observed between spring and summer, namely lower precipitation and intensification of northerly winds along the west coast (associated with upwelling events), and from the west along the south coast, and in autumn–winter, when precipitation is higher and there is a prevalence of southerly winds favorable to downwelling [41,42,43]. The productive period (based on Chlorophyll-a concentrations) is defined from April to September for Portuguese coastal waters [38]. River runoff has a significant impact on nutrient concentration in the coastal zone (greater on the W coast) and on stratification of the water column; especially along the NW coast where a greater number of coastal water bodies occurs compared to the remaining coastal areas [44]. Runoff may be associated with the formation of low-salinity buoyant plumes that impact biological fields due to their efficiency in retaining organic matter [45]. *Dinophysis acuminata* has been reported to be associated with these lenses, in particular growing within thin layers [46].

### 4.2. Sampling Strategy

The time series analyzed comprise seven years (2014 until 2020) of weekly sampling of water (with a bucket, if the water column had <5 m depth and with a hose if depth was >5 m) and shellfish from coastal harvesting areas (Figure 1), collected simultaneously from each harvesting area. Samples were routinely collected under the National System of Monitoring Shellfish (SNMB) for human consumption, held by IPMA, I.P. (Portuguese Institute for the Sea and Atmosphere) [39]. The sampling grid analyzed includes 18 coastal stations distributed along coastal shellfish harvesting areas.

Water samples were collected during high tide (±1 h) and preserved with 1% neutral Lugol’s iodine solution, in the field [21].

Regarding shellfish samples, since bivalves species have different distributions and abundances alongshore and with depth, sentinel species for the NW, SW, and S coasts were *Mytilus galloprovincialis* (intertidal/water column organism) and also *Donax trunculus* (substrate organism) for the SW and S coasts. For shellfish samples, the time series consisted of weekly sampling of the sentinel species for each coastal area during the seven-year period.

### 4.3. Cell Estimation

Samples were analyzed within 24–48 h of collection by settling 50 mL of Lugol preserved water following the Utermöhl (1958) method [47]. The samples were examined for the presence of all *Dinophysis* species including *D. acuminata*, with an inverted microscope equipped with phase contrast and bright field illumination (Leica DMi8), at a magnification of 200× with a detection limit of 20 cells L^−1^ (abundances were expressed in cells L^−1^).

### 4.4. Biotoxins Analysis

The bivalve soft tissues were removed from the shell, washed with running tap water to remove residues, drained and then homogenized in a blender. Toxin extraction was performed according to the standard operating procedure (SOP) for the determination of lipophilic marine biotoxins in molluscs by LC-MS/MS provided by the European Union Reference Laboratory [48,49]. Briefly, a 2 g portion of homogenized tissue was double-extracted with 100% methanol, followed by alkaline hydrolysis in order to determine the total content of OA group, by converting the acylated esters of OA and/or dinophysistoxins, DTX1 and DTX2 (DTXs), to the parent OA and/or DTXs. After hydrolysis, the extracts were filtered and analyzed by liquid chromatography with tandem mass spectrometric detection (LC-MS/MS). From 2014 to 2016 the LC-MS/MS analysis were performed with a Thermo Dionex Ultimate 3000 LC-system coupled to a Thermo TSQ Quantum Access Max triple quadrupole mass spectrometer, and after 2016 sample extracts were analyzed in an Agilent 1290 Infinity LC-system coupled to an Agilent 6470 triple quadrupole mass spectrometer. A six-point calibration curve of OA, DTX 1, and DTX2 with a correlation > 0.990 was developed for quantification, with a quantification limit of 28 µg OA equiv kg^−1^.

### 4.5. Model Selection

#### 4.5.1. Dataset Analysis

From 2014 to 2020 the dataset available was 6463 for cells in the water, 2581 for toxins in shellfish, and 512 zeros for both cells and toxins. Cells in the water (referred hereinafter only as cells) and toxins in shellfish (referred hereinafter only as toxins) are datasets that have a strong seasonality and a high degree of intrinsic variability. Data are over-dispersed and there are marked periods of presence versus absence (significant periods with zero concentrations determined for both cells and toxins). During the optimal period for the toxigenic *Dinophysis* species, February to November, cells are regularly found in monitoring samples but ambient conditions are not always conducive to the growth required to achieve warning concentrations (200 cells L^−1^ or 500 cells L^−1^). The persistence of cells in the water ≥ 100 cells L^−1^ for more than two weeks was weighed in the model. Regarding the toxins dataset, the contribution of DTX1 and DTX2 for the total content of the OA group were subtracted, since DTX1 was absent and the presence of DTX2 was associated with the presence of *D. acuta*. Concentrations above 400 µg OA kg^−1^ were discarded from the analysis since the focus was the closure level (160 µg OA equiv kg^−1^ or below) and its association with cells in the water at an early stage of development. High concentrations can lead to an excess of variability, making it difficult to isolate specific correlations at lower concentrations, where correlations relevant to advanced warning may be subtler. By removing high concentrations, our aim was to enhance the precision of the model, allowing for a more focused analysis on toxin vs. cell concentrations that are more pertinent to the goals of the study, thereby increasing confidence in the interpretation of the results obtained. These adjustments were focused on fine-tuning the model’s parameters to fit the training data and enhance the model’s ability to generate accurate predictions on the training data. We were also mindful of avoiding overfitting, ensuring that the model’s performance during validation was not compromised. The objective was for the model to excel in both training and new data scenarios.

The algorithm developed herein had two primary objectives: firstly, to determine the threshold concentration of *Dinophysis acuminata* in the water, capable of triggering the closure of harvesting areas due to the presence of biotoxins in *Mytilus galloprovincialis* and *Donax trunculus*, specifically at 160 µg OA equiv kg^−1^ of shellfish meat or edible product. Secondly, the algorithm aimed to assess the extent to which it is feasible to anticipate the toxification of these shellfish species, enabling the implementation of effective mitigation actions.

As described, the Portuguese western and southern coastlines exhibit distinctive geographic and oceanographic characteristics. The model was fitted spatially, for each coastal area (NW, SW and S) to evaluate how regionally an early warning threshold may need to be developed and for each bivalve sentinel species. Given that the existence of *Dinophysis* cells in water typically precedes the detection of biotoxins, lags of one and two weeks between cell and toxin detection were fitted. The fitting baseline of the model was the same, while changing the way the data are assumed to be distributed or the probability distribution that best describes them.

#### 4.5.2. Tested Models

For model validation, the seven-year dataset was partitioned randomly by computer with 75% used to fit the model and 25% used to validate model accuracy (*n* = 1616). The model predictive accuracy was calculated as well as the predictive *D. acuminata* concentrations that lead to harvesting bans with one- and two-week lags for both shellfish species. In accordance with the characteristics of the dataset and the assumptions above, four generalized additive models (GAMs) with distributions suitable for overdispersed or Zero inflation data (Poisson, Tweedie, Zero-inflated Poisson and Negative Binomial) were tested using the “mgcv” package of the R software (version 1.9-1) [50].

The concentration of *D. acuminata* (discrete variable) was used as a dependent variable and the predictive variables were (i) okadaic acid concentration per shellfish species by lag week (discrete variable) with a thin-plate smoothing spline having nine knots (k = 9), (ii) geographic zones (categorical variable with three levels), and (iii) the persistence or increase in *D. acuminata* cells in the water (categorical variable with two levels, “0” for persistence and “1” for increase). These predictive variables were selected following Wood (2001) [51]. Smoothness parameters were estimated via restricted maximum likelihood (REML).

The four models were tested to identify the best way to minimize the over dispersion and to better accommodate the excess of zeros in the data having the best fit possible. The model selected had the best compromise between having the lowest AIC scores and root mean squared error (RMSE) and the highest deviance explained and performance score (percentage of correct predictions). The predicted variables were plotted and the shape of the functional form indicates, if positive, that covariates are related positively to the dependent variables and vice versa, if negative, according to Wood (2006) [52].

## Figures and Tables

**Table 1:** Annual *Dinophysis acuminata* maxima (cells L^−1^) in the water and region and maximum concentrations (µg OA equiv kg^−1^) identified in mussel and donax samples per region. Concentrations exceeding 850, 625, and 550 µg OAO equiv kg^−1^ surpass the limits of the calibration range used for quantifying OA, DTX1, and DTX2. The parentheses contain an approximate reference regarding how many times the European Union Reference Level (EU RL) has been surpassed. The number of times the regulatory limit (RL), 160 µg OA equiv kg^−1^ of shellfish-derived edible product, was surpassed by shellfish species.

Region	Year						
(cells L^−1^)	2014	2015	2016	2017	2018	2019	2020
**NW**	7544	3180	4300	8140	4660	1740	12,960
**SW**	3700	3500	2240	820	580	1560	700
**S**	7200	2380	3740	280	3420	1700	11,660
(μg OA equiv/kg)	**2014**	**2015**	**2016**	**2017**	**2018**	**2019**	**2020**
**NW**	>850 (12 × RL)	>850 (13 × RL)	>850 (23 × RL)	>625 (37 × RL)	>550 (50 × RL)	>550 (8 × RL)	>550 (9 × RL)
**SW**	>850 (10 × RL)	>850 (17 × RL)	>850 (23 × RL)	>625 (13 × RL)	587	649	>550 (5 × RL)
**S**	1402	>850 (14 × RL)	>850 (10 × RL)	>625 (4 × RL)	>550 (8 × RL)	830	>550 (10 × RL)
Number of times RL was surpassed in *Mytillus*	**2014**	**2015**	**2016**	**2017**	**2018**	**2019**	**2020**
**NW**	72	68	41	40	20	58	52
**SW**	16	17	13	17	15	15	15
**S**	20	23	16	13	19	18	23
Number of times RL was surpassed in *Donax*	**2014**	**2015**	**2016**	**2017**	**2018**	**2019**	**2020**
**NW**	-	-	-	-	-	-	-
**SW**	25	22	20	25	20	19	17
**S**	43	36	25	23	14	11	16

**Table 2:** Spearman’s correlation (ρ) between D. acuminata and two different shellfish species, also tested with 1- and 2-week lags. Values where 0.20 ≤ |ρ| ≤ 0.29 indicate a weak monotonic association [29]. A positive value indicates a positive monotonic relationship.

Spearman Correlation	Shellfish	
	*Mytilus*	*Donax*
*D_acuminata*	0.23	0.26
Lag1_*acuminata*	0.27	0.36
Lag2_*acuminata*	0.26	0.34

**Table 3:** Results of model fitting (performance score) and model validation (accuracy) with different GAM distribution and their respective quality criteria. Model TW-GAM performed best. GAM: generalized additive model, GAMTW: Tweedie generalized additive model, NBGAM: negative binomial generalized additive model, ZGAM: zero-inflated generalized additive model, GAMPOIS: Poisson generalized additive model, AIC: Akaike information criterion, RMSE—root mean square error.

			Model Fitting					Model Accuracy (%)
Species	Lag	Distribution	Deviance Explained	AIC	RMSE	*n*	Performance Score (%)	
*Mytillus galloprovincialis*	Lag1	**Tweedie**	**68.7**	**3351**	**1.43**	1011	**86.46**	**79.4**
		Poisson	75.4	36471	2.74		59.55	8.1
		Z-Inflated	83.7	17121	0.74		51.14	79.4
		Negative Binomial	23.6	3754	147		20.00	79.4
	Lag2	**Tweedie**	**68.1**	**3391**	**1.43**	1009	**86.47**	**78.7**
		Poisson	75.5	38004	2.68		59.54	78.7
		Z-Inflated	92.7	16606	0.66		53.04	78.7
		Negative binomial	24.1	3805	137.49		20.00	78.7
*Donax trunculus*	Lag1	**Tweedie**	**69.00**	**1385**	**1.33**	279	**85.98**	**54.3**
		Poisson	81.9	10814	3.24		59.14	54.3
		Z-Inflated	100.00	5724	1.04		48.63	54.3
		Negative Binomial	28.3	1590			20.00	54.3
	Lag2	**Tweedie**	**64.4**	**1455**	**1.34**	278	**87.86**	**60.0**
		Poisson	76.1	13943	2.60		59.4	8.8
		Z-Inflated	100.0	7411	1.00		47.68	8.8
		Negative Binomial	27.6	1651	85.26		20.00	60.0

## Data Availability

No new data were created or analyzed in this study. Data sharing is not applicable to this article.

## Supplementary Materials

The following supporting information can be downloaded at: https://www.mdpi.com/article/10.3390/toxins16050204/s1, Figures S1–S4—GAM check for *Mytillus* and *Donax*, for 1- and 2-week lags. Figures S5–S8—Smooth plots for *Mytillus* and *Donax*, for 1- and 2-week lags.
