# Supplementary figures and images for: Advances in the Early Warning of Shellfish Toxification by Dinophysis acuminata
Source: Toxins (Basel). 2024 Apr 24;16(5):204. doi: 10.3390/toxins16050204 (PMC11126001; doi:10.3390/toxins16050204)

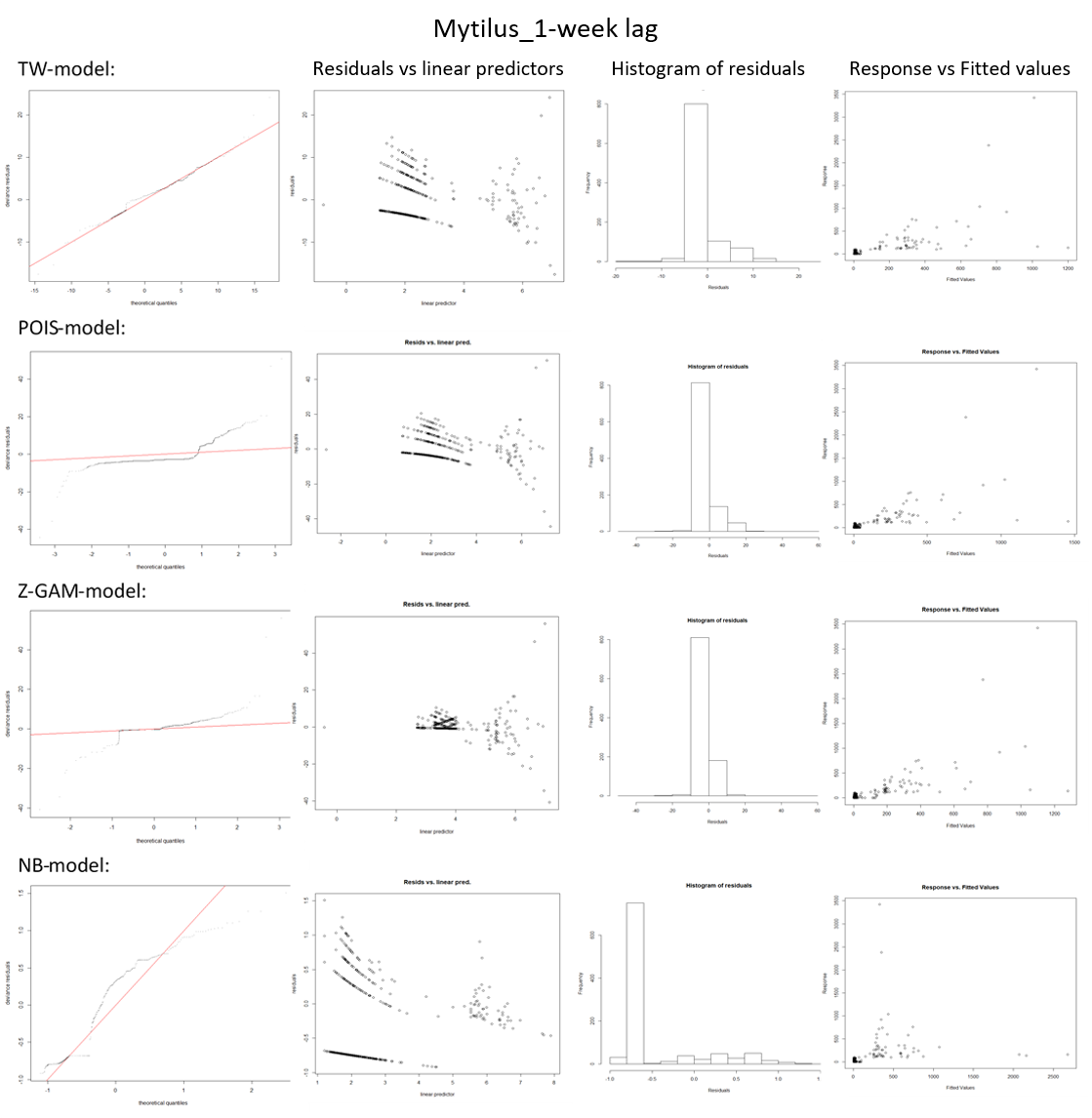

Supplement: Supplementary file 1 [file toxins-16-00204-s001.zip › Supp 1 - GAM-check Mytilus_1w-lag.tif]

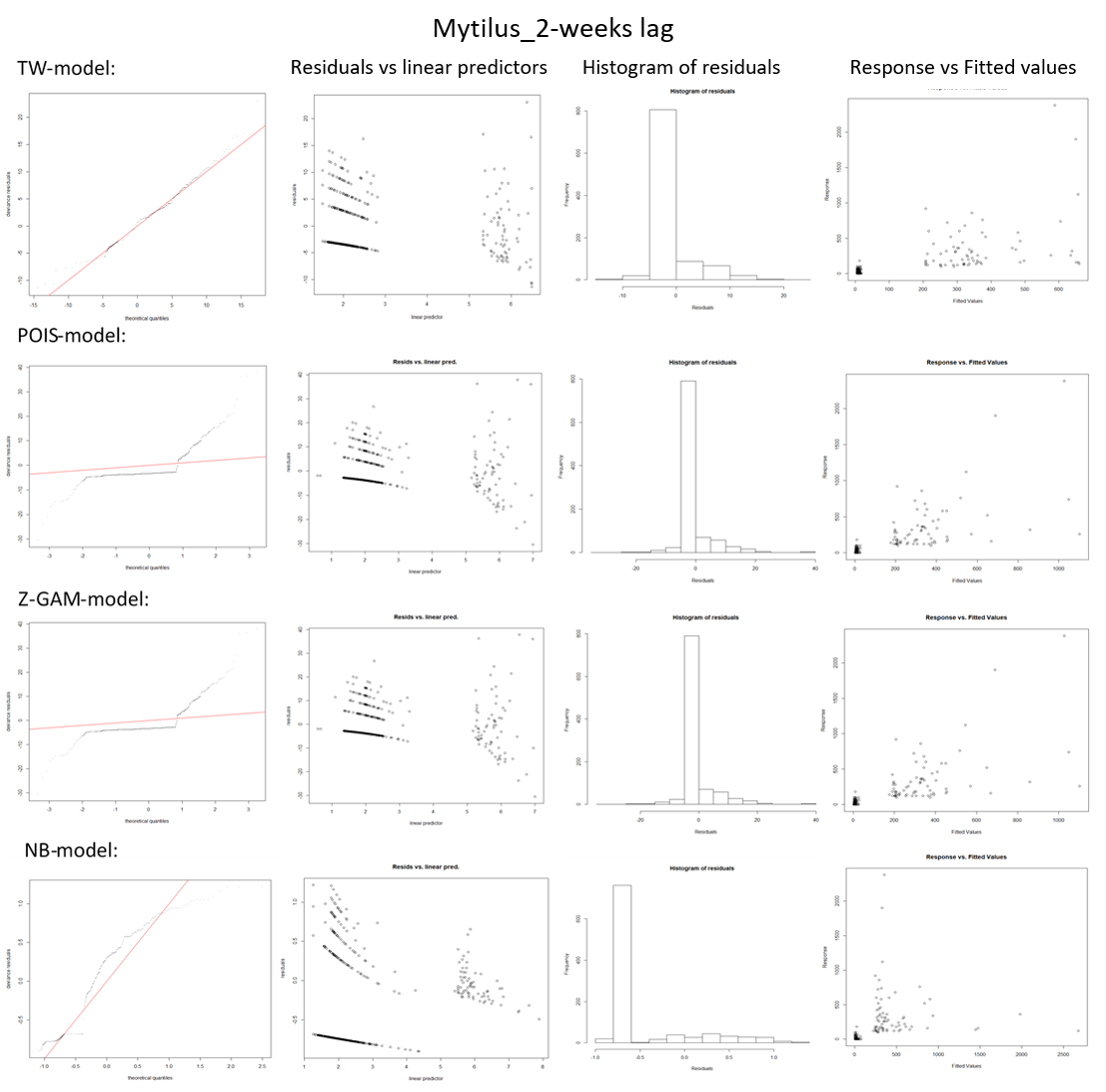

Supplement: Supplementary file 1 [file toxins-16-00204-s001.zip › Supp 2 - GAM-check Mytilus_2w-lag.tif]

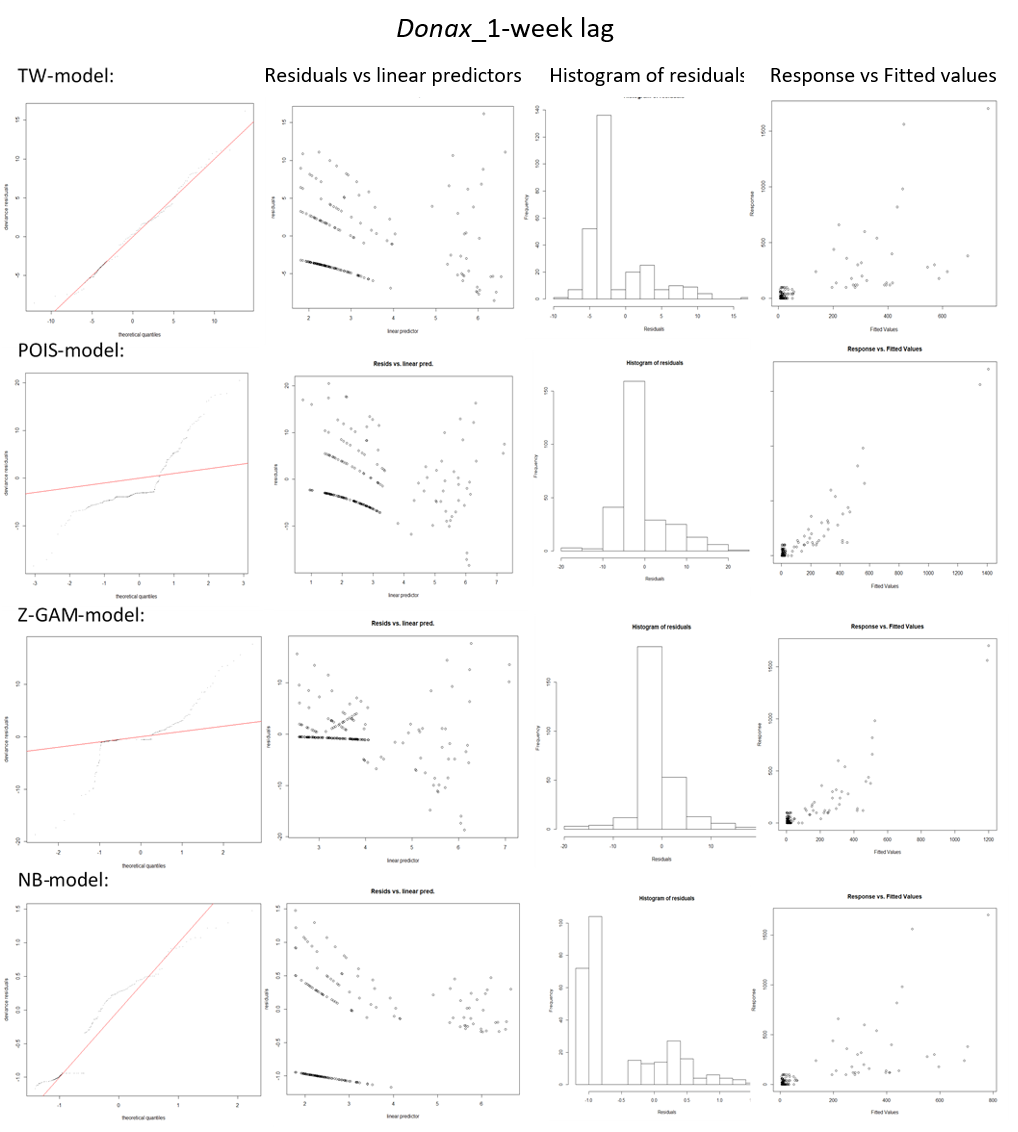

Supplement: Supplementary file 1 [file toxins-16-00204-s001.zip › Supp 3 - GAM-check Donax_1w-lag.tif]

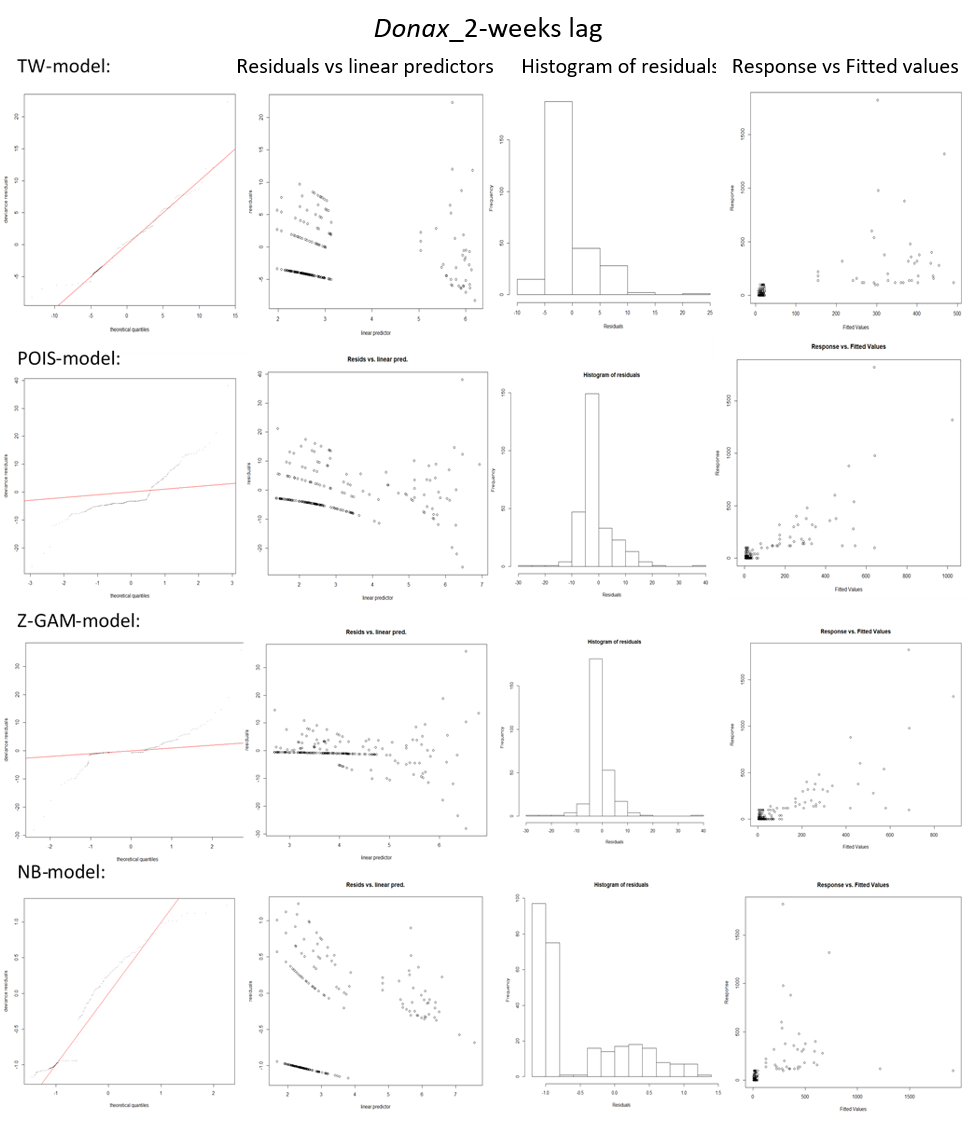

Supplement: Supplementary file 1 [file toxins-16-00204-s001.zip › Supp 4 - GAM-check Donax_2w-lag.tif]

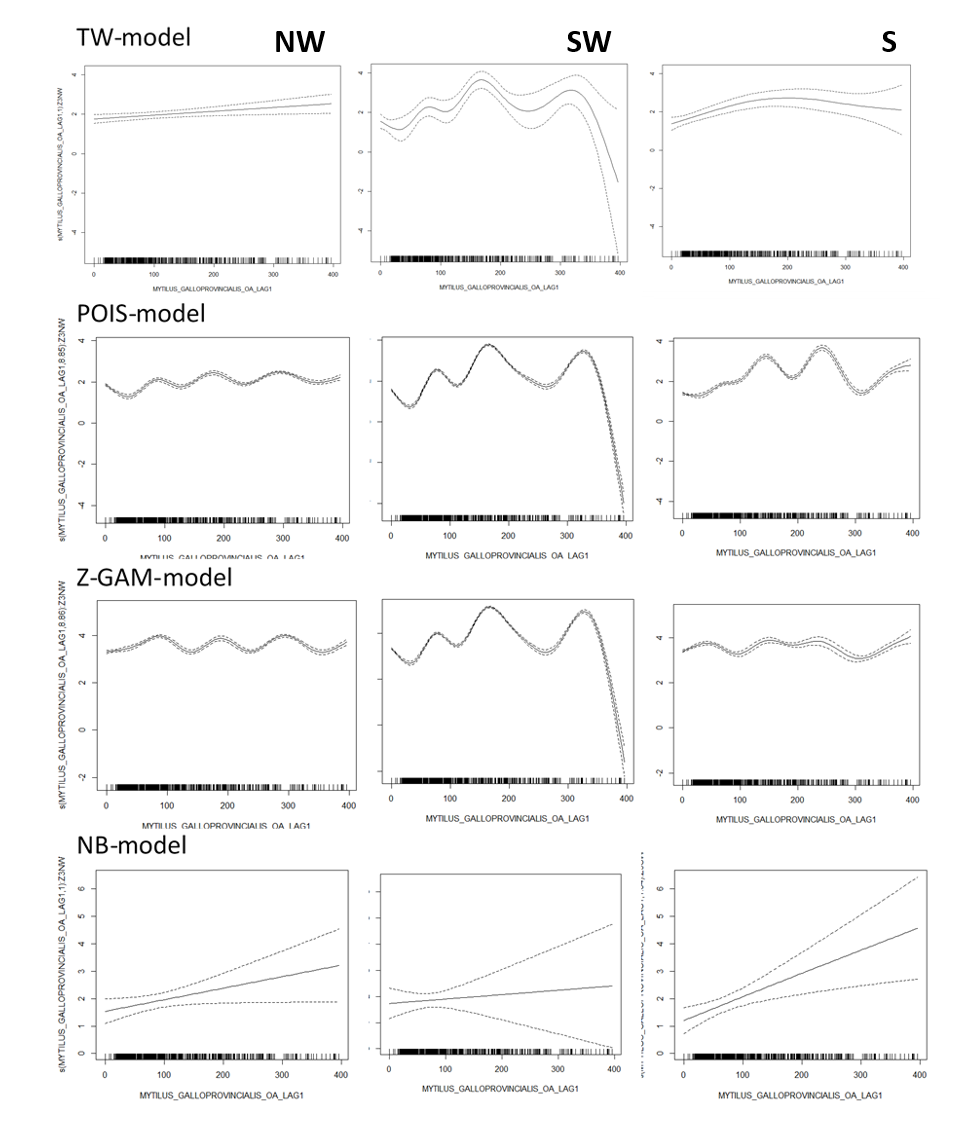

Supplement: Supplementary file 1 [file toxins-16-00204-s001.zip › Supp 5 - Smoth Mytilus_1w-lag.tif]

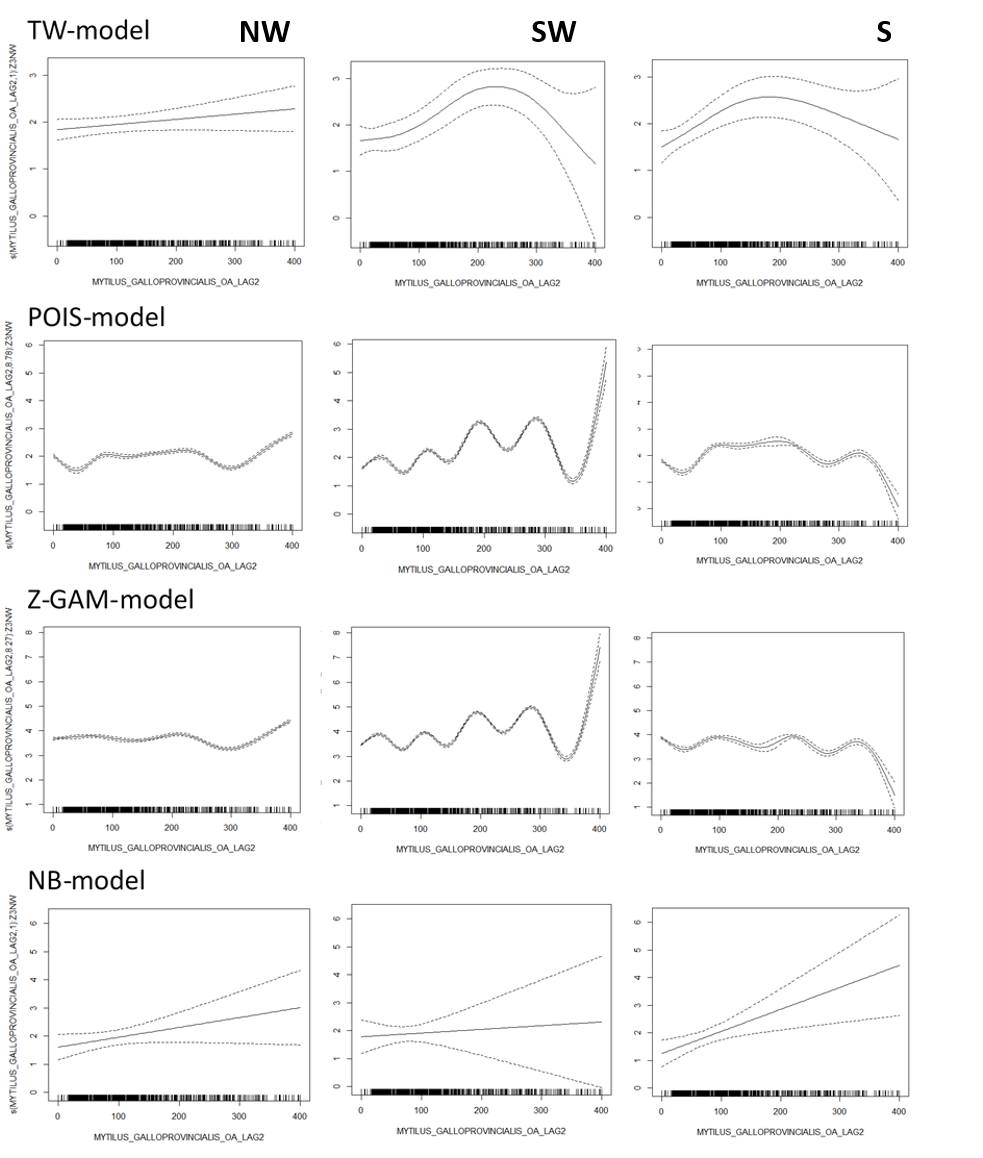

Supplement: Supplementary file 1 [file toxins-16-00204-s001.zip › Supp 6 - Smoth_Mytilus_2w-lag.tif]

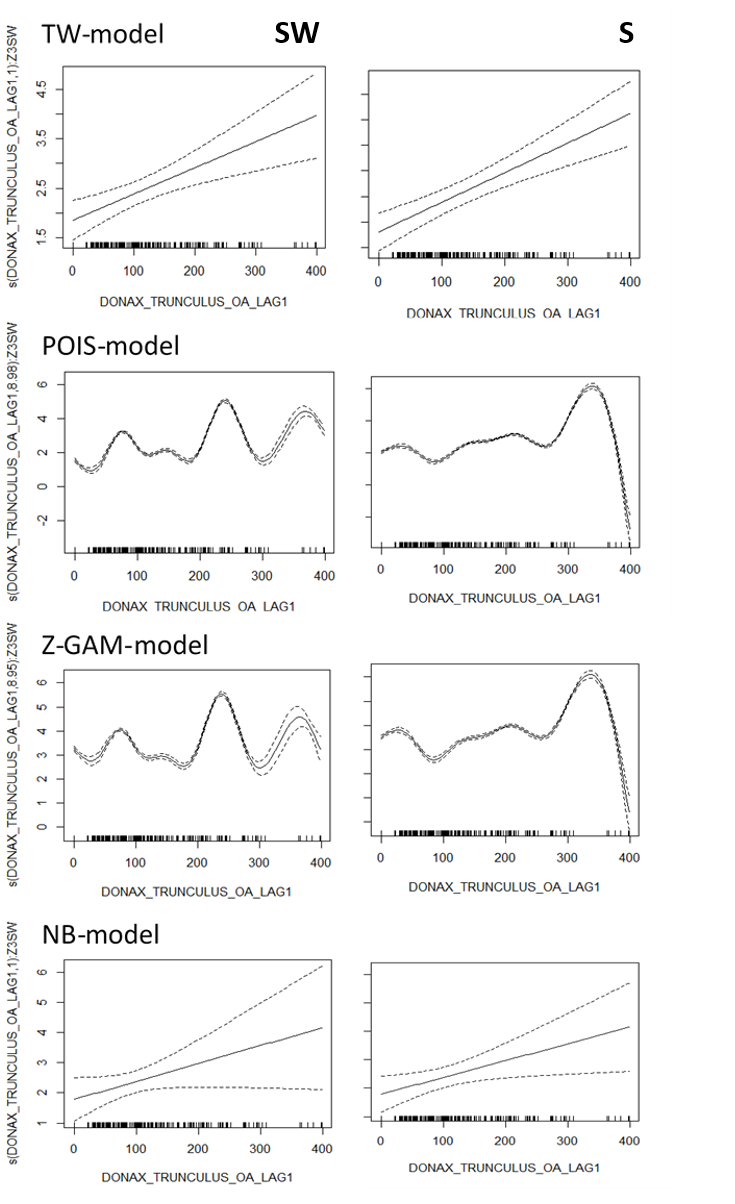

Supplement: Supplementary file 1 [file toxins-16-00204-s001.zip › Supp 7 - smooth Donax_1w-lag.tif]

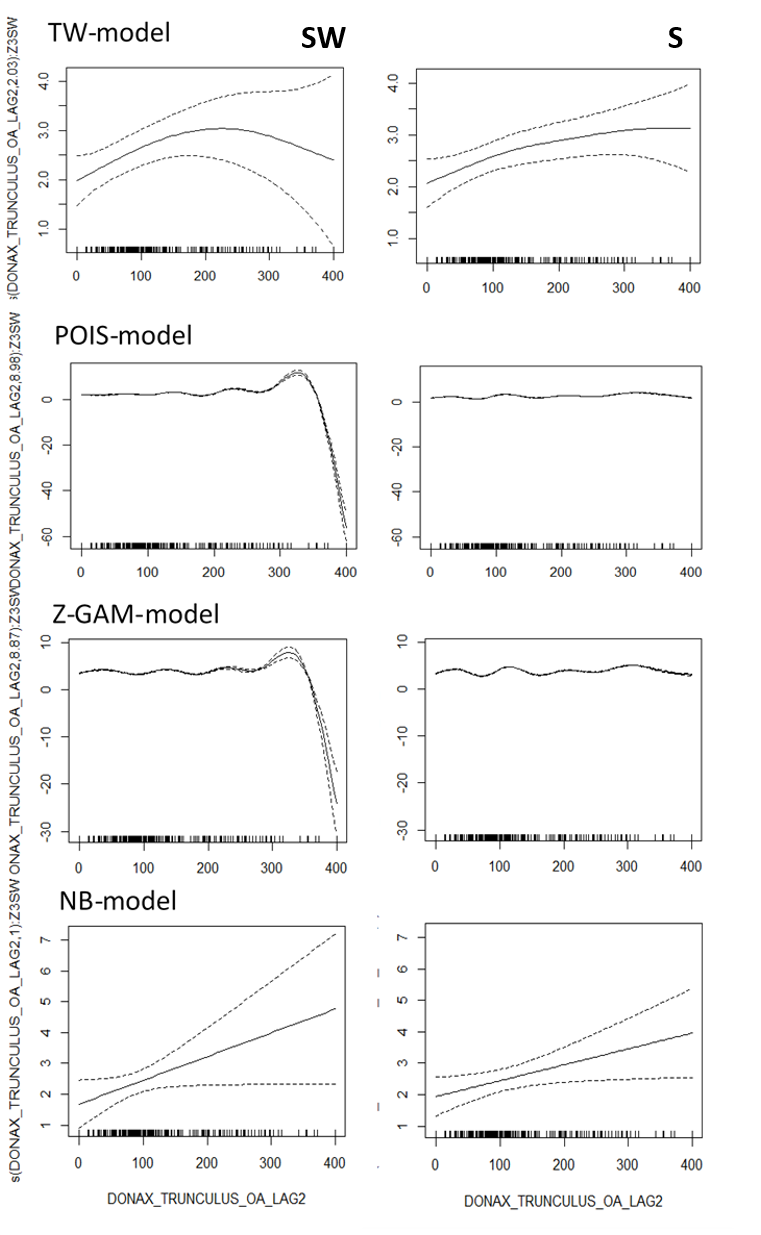

Supplement: Supplementary file 1 [file toxins-16-00204-s001.zip › Supp 8 - Smoth Donax_2w-lag.tif]
